# Effect of hydrochar-doping on the performance of carbon felt as anodic electrode in microbial fuel cells

**DOI:** 10.1007/s11356-024-33338-2

**Published:** 2024-04-23

**Authors:** Yelitza Delgado, Natalia Tapia, Martín Muñoz-Morales, Álvaro Ramirez, Javier Llanos, Ignacio Vargas, Francisco Jesús Fernández-Morales

**Affiliations:** 1 Department of Chemical Engineering, ITQUIMA, University of Castilla La Mancha, Campus Universitario S/N., 13071 Ciudad Real, Spain; 2 Department of Hydraulic and Environmental Engineering, Pontificia Universidad Católica de Chile, 7820436 Santiago, Chile

**Keywords:** Hydrothermal carbonization, Hydrochar, Electrodes, Microbial fuel cell, Electrochemical performance

## Abstract

In this study, the feasibility of using hydrochars as anodic doping materials in microbial fuel cells (MFCs) was investigated. The feedstock used for hydrochar synthesis was metal-polluted plant biomass from an abandoned mining site. The hydrochar obtained was activated by pyrolysis at 500 °C in N_2_ atmosphere. Under steady state conditions, the current exerted by the MFCs, as well as the cyclic voltammetry and polarization curves, showed that the activated hydrochar-doped anodes exhibited the best performance in terms of power and current density generation, 0.055 mW/cm^2^ and 0.15 mA/cm^2^, respectively. These values were approximately 30% higher than those achieved with non-doped or doped with non-activated hydrochar anodes which can be explained by the highly graphitic carbonaceous structures obtained during the hydrochar activation that reduced the internal resistance of the system. These results suggest that the activated hydrochar materials could significantly enhance the electrochemical performance of bioelectrochemical systems. Moreover, this integration will not only enhance the energy generated by MFCs, but also valorize metal polluted plant biomass within the frame of the circular economy.

**Supplementary Information:**

The online version contains supplementary material available at 10.1007/s11356-024-33338-2.

## Introduction

With the global shift towards a sustainable society, there is an increasing demand for renewable energy sources (Gude 2015; Krastev and Falcucci 2019). Microbial fuel cell (MFC) technology emerges as a viable solution, capable of generating electrical current through redox reactions of chemical compounds. Notably, these chemical compounds can be even pollutants contained in waste effluents (Bose et al. 2018). To achieve the chemical to electrical energy conversion, MFC technology integrates reduction reactions at the cathode with oxidation reactions at the anode, assisted with electrochemically active microorganisms, resulting in a voltage drop and consequent electrical current generation (Mateo et al. 2018a). In this sense, MFC technology not only facilitates energy recovery but also could act as a waste treatment technology by removing chemical pollutants (Xie et al. 2011; Gajda et al. 2018).

Nowadays, the major drawback of the MFC technology is the use of expensive electrodes (Xie et al. 2011), which makes this technology less competitive (Gajda et al. 2018). In the literature, it has been stated that he costs of conventional MFCs’ electrodes based on carbon paper or carbon cloth electrodes present prices ranging from 100,000 to 500,000 US$ per square meter. Electrodic materials such as granular activated carbon or granular graphite electrodes present average prices ranging from 500 to 2500 US$ per ton, which is significantly lower than that of the carbon paper or carbon cloth electrodes but is still considered high for large-scale applications. Hydrochar, however, is a more cost-competitive option with prices ranging from 51 to 381 US$ per ton, making this material very competitive from the economical point of view (Huggins et al. 2014). In the literature, the use of hydrochars from waste biomass have been described as an interesting option due to its high porosity, biocompatibility, chemical stability, durability, and high electrical conductivity (Cai et al. 2020; Ahanchi et al. 2022). These characteristics facilitate the biofilm development as well as the electron transfer, enhancing the bioelectrochemical performance of the MFC.

Parallel, soil pollution associated with abandoned mining sites represents a major environmental problem (Rodríguez et al. 2022; Sanchez-Ramos et al. 2023). In such soils, autochthonous plants accumulate high concentrations of metals (Zhang et al. 2020). Given the high metal content, the plant biomass is considered a toxic waste that requires proper management. The development of a technology that converts this waste into a valuable resource holds great significance and aligns with the current promotion of the circular economy concept (Leon-Fernandez et al. 2021a). Conventional thermal treatments like pyrolysis have been employed for decades. Unfortunately, their intensive energy demand and the need for raw material pre-treatment make them unattractive. Hydrothermal carbonization (HTC) emerges as one of the most sustainable alternatives for processing plant biomass (Kambo and Dutta 2015). HTC involves the conversion of biomass into other carbonaceous compounds in hot compressed liquid water, at a low temperature range, typically between 180 and 250 °C, and at elevated autogenous pressure to avoid the high energy costs of thermal treatments performed at approximately 900 °C (Fernández-Sanromán et al. 2021). Under these conditions, water remains in a subcritical state and facilitates a cascade of simultaneous reactions (Kambo and Dutta 2015). HTC processes different raw materials, generating a wide spectrum of product characteristics (Patwardhan et al. 2022). Hydrochar is a carbon-rich solid with high porosity and chemical functionality, which can reduce the mass and charge transfer limitations facilitating the electrochemical processes (Liu et al. 2020a, b) and, therefore, making this material very useful as electrode in bioelectrochemical systems (Hu et al. 2010; Zhu et al. 2014; Patwardhan et al. 2022). Additionally, the presence of metals in polluted plant biomass growing in abandoned mining sites (Medina-Díaz et al. 2022) can enhance the electrochemical properties of the hydrochar (Huggins et al. 2014). Therefore, it can be assumed that the application of hydrochar as doping material in MFC electrodes could enhance the performance of this technology.

In this context, this study explores the feasibility of using hydrochar, obtained from polluted plant biomass, as a doping material in MFC’s electrodes. The study mainly focuses on the electrochemical performance of the MFCs.

## Materials and methods

### HTC synthesis and electrode-doping

The feedstock used for the HTC process was metal-polluted plant biomass of *Spergularia rubra*, a picture of this plant, and the HTC process applied, which are presented in the supporting information (Fig. S1). *Spergularia rubra* plants naturally growing at the San Quintin mining site (Ciudad Real, Spain) are polluted with approximately 100–300 g/kg of Zn and Pb and approximately 10 mg/kg of Cu due to the pollution of the soil and water in this mining environment (Rodríguez et al. 2022).

The operational parameters for HTC processing were tailored to the characteristics of the *S. rubra* plant biomass. To ensure proper wettability and sample homogeneity the plant was crushed and a mixture of 100 g solid·L^−1^ pure water was used. The processing temperature, 200 °C for 2 h, was chosen due to significant mass loss observed in previous experiments at this temperature. To evaluate not only hydrochar-doping but also the influence of the activation process on the electrochemical performance of hydrochar-doping, the hydrochar was subjected to pyrolysis at 500 °C in nitrogen gas (flow rate of 0.5 L/min) to ensure inert conditions. Activated and non-activated hydrochars were used to dope the carbon felt (CF) used as anode in the MFC.

To dope the CF electrodes, an immersion deposition process previously described in literature was applied (Gonzalez del Campo et al. 2014). To achieve this, the CF electrodes were subjected to a cleaning pre-treatment. This pre-treatment was performed by immersing the electrode in acetone for 15 min under ultrasound, another 15 min in Milli-Q water under ultrasound, both at a frequency of 80 kHz, and finally in Milli-Q water for rinsing. The ink used for doping was prepared with 20 mL of isopropanol, 20 mg of hydrochar (non-activated or activated), and 1 g of polytetrafluoroethylene solution (PTFE). The ink was subjected to ultrasound radiation for 30 min to facilitate homogenization. Once the ink was synthesized and homogenized, the CF electrodes were immersed in it and subjected to ultrasound at a frequency of 80 kHz for another 30 min. Subsequently, the electrodes were dried in a muffle furnace at 80 °C for 24 h (Ramírez et al. 2023). Thereafter, two different electrodes were obtained: a CF electrode doped with non-activated hydrochar (CFnaH) and a CF electrode doped with activated hydrochar (CFaH). Both electrodes had a doping load approximately 40 mg/cm^2^ of hydrochar. A raw CF electrode was used as a reference to evaluate the enhancement of the electrode-doping.

### Microbial fuel cell

The MFC setup used in this study consisted of MFCs with separate compartments. To study the effect of hydrochar doping on the electrochemical performance of MFCs, the following configurations were studied: MFC operating with a raw CF anode, MFC operating with a CF anode doped with non-activated hydrochar, and MFC operating with a CF anode doped with activated hydrochar. Parallel abiotic experiments were conducted to isolate chemical processes from the biological processes caused by the planktonic and biofilm cultures developed in the biotic setups. All the experiments were performed in duplicate to ensure reproducibility. All the MFC systems used in this study were composed of 100-mL anodic and cathodic chambers. These compartments were separated using a bipolar membrane (Fumasep® FBM) and connected using an external electrical circuit loaded with a resistance of 120 Ω (González Del Campo et al. 2016). The MFCs were made of transparent PVC and sealed with silicon gaskets to prevent liquid leakage from the anodic and cathodic compartments. The anodes were made of CF (KFA10, SGL Carbon Group), dimensions 2.5 × 2.5 × 0.8 cm, with or without doping agents. CF was selected because it has been described in the literature as a carbonaceous material with excellent electrochemical performance (Mateo et al. 2018c). Moreover, its porous structure facilitates biofilm development, which enhances its bioelectrochemical performance (Mateo et al. 2018d, c). The cathode was composed of titanium, 2.5 × 2.5 cm. Titanium was used as the cathodic material because of its mechanical resistance and electrochemical performance. To avoid fluctuations due to changes in the operational conditions, all the MFCs were operated at 25 °C.

For the start-up of the MFC, the anode was filled with 50% activated sludge and 50% synthetic wastewater, which was composed by 1 g·L^−1^ CH_3_COONa, 3 g·L^−1^ Na_2_HPO_4_, 0.7 g·L^−1^ KH_2_PO_4_, 0.8 g·L^−1^ (NH_4_)_2_SO_4_, 0.2 g·L^−1^ MgCl_2_·6H_2_O, 0.05 g·L^−1^ CaCl_2_, and 0.04 g·L^−1^ (NH_4_)_2_Fe(SO_4_)_2_·6H_2_O. It has been reported that acetate promotes biofilm growth and energy production; thus, it is generally used as a carbon source in MFCs (Mateo et al. 2018b). The fresh anodic medium had a pH of 7 and conductivity of 4.27 mS·cm^−1^. The activated sludge used as the inoculum was obtained from the domestic wastewater treatment plant of Ciudad Real; more information about this facility can be found elsewhere (Rodríguez Mayor et al. 2004).

Simultaneously, the catholyte was filled with a supporting electrolyte according to the literature (Leon-Fernandez et al. 2021b). The catholyte was composed of 4 g·L^−1^ Na_2_SO_4_ and was continuously aerated using an air pump connected to a diffuser that generated microbubbles. Electrogenic microbial culture development required approximately 250 h. To ensure the availability of biodegradable substrates at the anode, the anolyte was replaced every 2 days with fresh medium.

### Analytical methods

A GLP22 Crison pH meter was used to measure pH. The biodegradable substrate concentration at the anode was determined by measuring the chemical oxygen demand (COD) according to a procedure described in the literature (American Public Health Association EADAWWAWEF 2005). The soluble COD concentration was determined to avoid interference from detached or planktonic biomass. To do this, samples were filtered through 0.22-µm glass fiber filters (De Lucas et al. 2007). Filtered samples were subjected to strongly oxidation conditions with a known excess of potassium dichromate (K_2_Cr_2_O_7_). After digestion, the remaining unreduced K_2_Cr_2_O_7_ was analyzed to determine the amount of K_2_Cr_2_O_7_ consumed and the oxidizable matter was calculated in terms of oxygen equivalent.

Determination of HTC intermediates was also performed from filtered samples by means of an HPLC (Agilent Technologies) with an UV–DAD detector at a detention wavelength of 210/8 nm, reference wavelength of 360/80 nm, and Zorbax SB-Aq (4.6 × 150 mm 5 µm) column. The oven temperature was 25 °C, and the mobile phase was a buffer solution of 20 mM of NaH_2_PO_4_ and H_3_PO_4_ (Fernández-Morales et al. 2010). Calibration was performed each time the mobile phase was replaced. Raman spectra were recorded in ambient conditions in a Renishaw InVia Qontor spectrometer equipped with 514.5-nm laser. The spectra were collected under a Leica DM2500 optical microscope with a × 50 long working distance objective (approximately 10 mm). The scattered Raman light was dispersed by a holographic grating of 600 grooves/mm, in order to acquire the whole range of interest for carbons (500–5000 cm^−1^). Hydrochars were characterized using High-Resolution Scanning Electron Microscopy (HRSEM) coupled with Energy-Dispersive X-ray Spectroscopy (EDS) detector using a ZEISS GeminiSEM 500 Microscope. X-ray diffraction measurements were realized by a Philips X’PertMPD with Kα radiation from copper radiation, *λ* = 1.54056 Å, with graphite monochromator and xenon gas sealed detector. Operation conditions were 2 theta angles between 3 and 100° with a sweep speed of 0.05° s^−1^. Surface functional groups and aromatic groups of carbon materials in the range of 2500 to 400 cm^−1^ was determined by an Agilent Cary 630 FTIR spectrometer. For thermogravimetric analysis (TG and DTG), the Q50 TGA model (TA Instruments, New Castle, EE.UU.) was used. Stabilization was performed at 100 °C for 20 min to remove all moisture, after which TG curves were obtained from 100 to 800 °C at a heating rate of 10 °C min^−1^ in an inert atmosphere (constant flow rate of 100 mL min^−1^ for N_2_ gas) (Castro et al. 2021a). The electrical conductivities of the activated and non-activated materials were measured using the four-point probe method according to ASTM standard method D4496-87. Pellets were prepared with 90 wt% carbon material, 10 wt% 1-methyl-2-pyrrolidinone, and a drop of PVDF as a binder, as previously reported (Casanova et al. 2020).

### Electrochemical methods

The electrical current exerted by each MFC was measured online every minute using a Keithley 2000 multimeter. KickStart software for online data storage was used to record electrical current data. Additional information on the setup used in this study can be found elsewhere (Leon-Fernandez et al. 2021a). Polarization curves and cyclic voltammetry were determined weekly using an NEV3 Potentiostat (Nanoelectra S.L., Spain) at a scan rate of 0.001 V s^−1^ from the OCV to 0.001 V in the case of the polarization curves, and at a scan rate of 0.001 V s^−1^ and a step potential of 0.005 V in the case of cyclic voltammetry (Villaseñor Camacho et al. 2017). The scan ranged from − 0.9 to 0.9 V, starting the scans at a mid-point of 0.005. Ag/AgCl was used as the reference electrode.

## Results and discussion

### HTC characterization

**Fig. 1:**
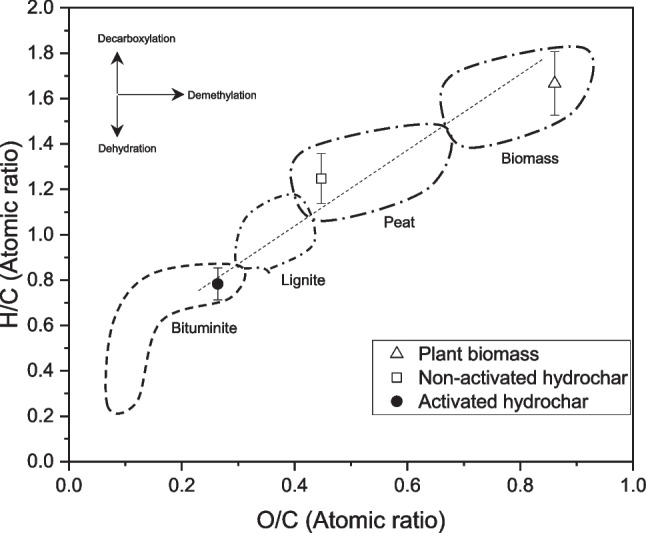
Van Krevelen diagram of the materials used in this study

In this study, hydrothermal carbonization (HTC) was selected as the treatment method for re-using plant biomass wastes, specifically *S. rubra*. This choice was motivated by the method’s low energy requirements and its versatile processing conditions when compared to torrefaction or pyrolysis that requires low humidity or higher processing times (Hoekman et al. 2017). The primary objective of HTC was to transform metal-polluted *S. rubra* plant biomass into hydrochar with enhanced electrochemical and physicochemical properties. Figure 1 illustrates the ultimate analysis in the Van Krevelen diagram comparing *S. rubra* plant biomass with the two hydrochars obtained after HTC: non-activated and activated hydrochars. As mentioned previously, the obtained hydrochar was activated through a pyrolysis process at 500 °C in an inert atmosphere.

In Fig. 1, non-activated and activated hydrochars showed lower H/C and O/C ratios compared to the raw *S. rubra* biomass. The condensation reactions give rise to the formation of carbonaceous structures, with the release of gas products such as CO, CO_2_, H_2_, and CH_4_ during the hydrothermal process, as previously reported elsewhere (Kambo and Dutta 2015).

The reductions in the H/C and O/C ratios can be attributed to dehydration and decarboxylation reactions occurring during the hydrochar synthesis via HTC. Concerning the pyrolysis (activated hydrochar), polymerization reactions were induced, leading to the degradation of structural components of the plant biomass, such as hemicellulose, cellulose, and lignin, in alignment with existing literature (Azzaz et al. 2020). Presence of metallic ions and heavy metals have influence in their thermal behavior and structural and electrochemical properties because it can reduce the temperature required to graphitize the carbon and could increase electric conductivity. In both samples specially in the activated hydrochar detectable concentration of Fe, Zn, and Pb and an incipient porous structure were found (Fig. S2). However, main factors related to the increase of microporosity and mesoporosity and electrical conductivity are the degradation of biomass during HTC. These transformations are crucial for improving external surface availability and consequently reducing potential mass transfer limitations. Furthermore, this process led to a higher carbon content in the hydrochar samples.

**Fig. 2:**
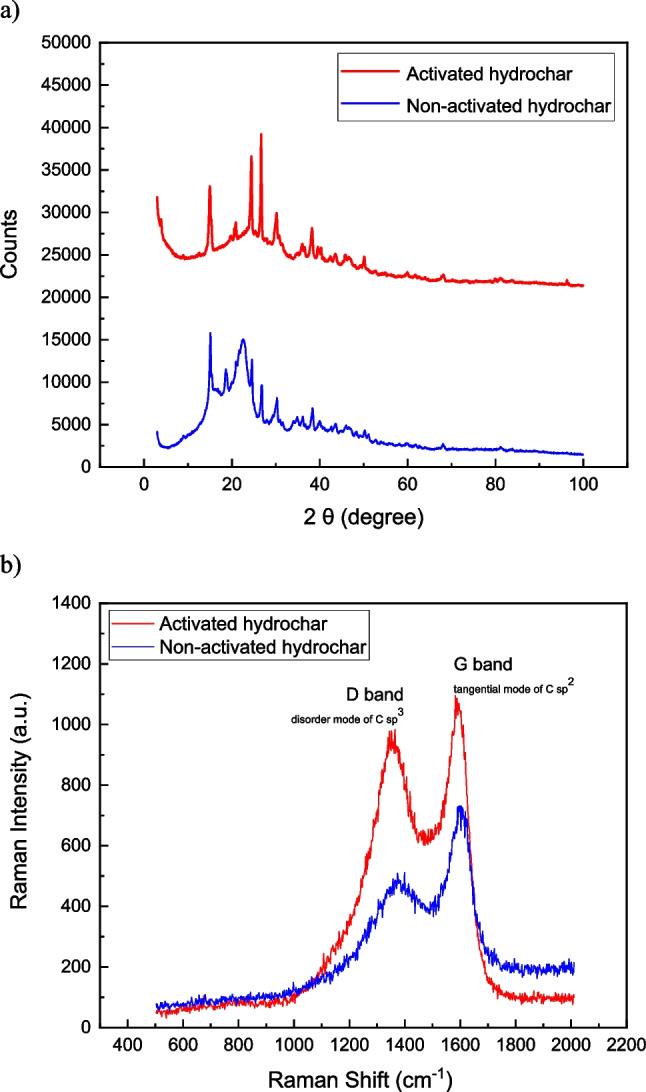
**a** XRD analysis of both hydrochars dopped on anodes before the MFC operation. **b** Raman spectra of both hydrochars studied

To carry out the structural characterization of the carbon materials, carbon ordering, and crystallinity, XRD analysis was carried out. XRD patterns of the materials are presented in Fig. 2(a). Most of the peaks obtained are near 25° corresponding to the crystal plane index C (002), which, in turn, is related to the parallel and azimuthal orientation of the aromatic and carbonized structure. The sharp peaks observed indicate a high degree of orientation. Moreover, the symmetry indicates the absence of γ-bands linked to amorphous and aliphatic structures (Lu et al. 2002). The XRD results show that biochar obtained at 500 °C is highly graphitic, which is desirable for effective electrochemical performance. As the pyrolysis was carried out at 500 °C, the characteristic diffraction peaks at 25° shift towards the left, which indicate an increase of structural order and graphitization of the sample (Mulyadi et al. 2017). Also, the high number of small peaks are related with the presence of iron salts and copper compounds adsorbed during the previous metal accumulation processes carried out by this plant biomass. Similar peaks related to Fe and Cu were found in electrodes during a Cu electrodeposition (Delgado et al. 2023).

Microstructural characteristics of the catalysts were determined through Raman Spectroscopy and CO_2_ adsorption isotherms. The Raman spectrum presented the typical shape of char materials due to the presence of polycyclic aromatic hydrocarbons (Yamauchi and Kurimoto 2003; Sevilla and Fuertes 2009; McDonald-Wharry et al. 2013). The peaks G and D are characteristic of graphitic materials and are due to the C–C stretching and aromatic breathing mode, respectively (Tuinstra F. and Koenig J. L. 1970). The D mode is defect-induced and is used as an indicator of the degree of crystalline order in graphene-like materials. The ID/IG peak intensity ratio has a value of 0.9 in the case of the activated hydrochar whereas its value was 0.7 in the case of non-activated hydrochar. The Raman spectra (Fig. 2(b)) revealed structural and in-plane defects in both hydrochars according to two pronounced characteristic peaks at 1350 cm^−1^ (D band, indicating defects in graphite) and 1590 cm^−1^ (G band, signifying planar vibrations of sp^2^ graphitic carbon) (Kumar et al. 2011; Sevilla et al. 2011). Thus, low D band indicates high structural order with high size aromatic domains associate to low density of structural defects that would reduce the ohmic losses. In activated hydrochar, peaks are narrower and more defined which suggest a better electrochemical behavior of MFC anodes. From these results it was confirmed a typical graphitic Raman spectrum which is comprised a higher G band peak and a lower D band peak (Dreyer et al. 2010; Chen et al. 2012; Zhang et al. 2013).

**Fig. 3:**
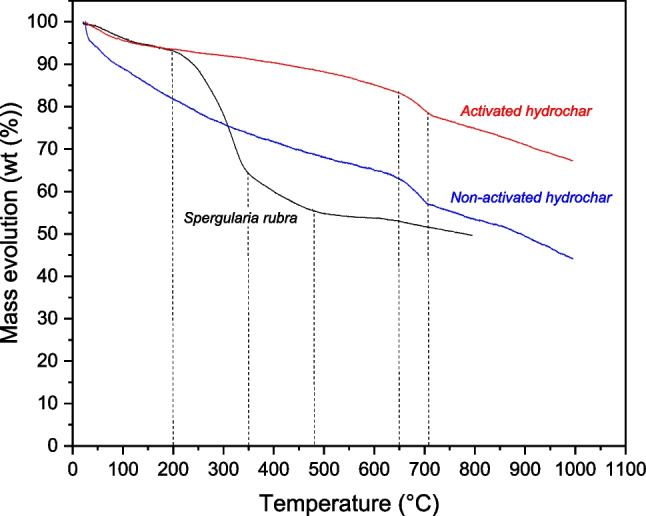
Thermogravimetric curves corresponding to *Spergularia rubra* plant biomass, non-activated hydrochar, and pyrolyzed hydrochar at 500 °C

Structural stability and combustion characteristics of hydrochars were evaluated through a thermogravimetric analysis. The results obtained from this analysis, in the form of mass loss percentages, are presented in Fig. 3.

Gravimetric analysis indicates that *Spergularia rubra* biomass initiates combustion at about 201 °C and ends at 342 °C, having the highest peak of the curve at 310 °C, which is located in the range of carbonization temperatures for hemicellulose and cellulose (Parshetti et al. 2013). It can also be appreciated a small curve corresponding to lignin, above 400 °C, probably due to low percentage of lignin from the biomass (Harmsen et al. 2010).

**Fig. 4:**
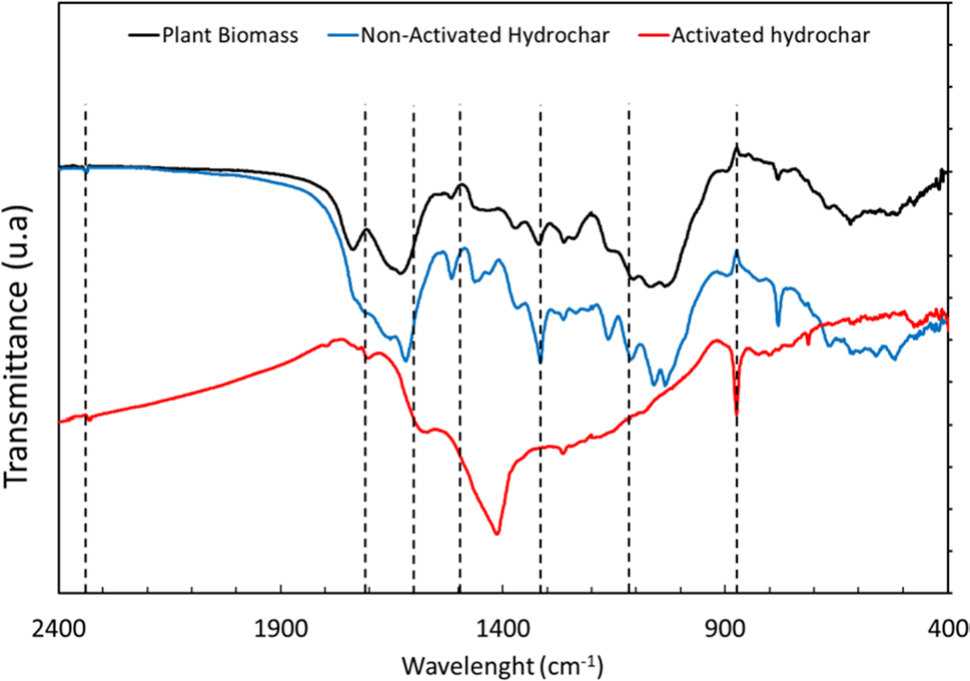
FTIR analysis of plant biomass and hydrochars used to modify carbon felt surface

Thermogravimetric analysis revealed that the stability drastically increased when the samples were subjected to pyrolysis at 500 °C. This can be verified by analyzing Fig. 3, where the mass loss of the *S*. *rubra* biomass exceeded that of the non-activated hydrochar, and the latter was higher than that of the activated hydrochar. HTC promotes dehydration and probably dehydroxylation, generating volatile organic compounds that enter the liquid phase. These reactions modify the chemical structure of the hydrochar, synthesizing new components that improve the homogeneity of samples (Funke and Ziegler 2010). Regarding to the activated and non-activated hydrochars, it is important to highlight the importance of the weight loss experienced at 660 °C. This weight loss was observed in both activated and non-activated hydrochars, and could be explained by the decomposition of the inorganic components in the ashes accumulated during the pyrolysis (Liu et al. 2012). The weight losses of the raw plant biomass and non-activated hydrochar exceeded 30%. However, in the case of the pyrolyzed hydrochars, a significantly lower weight loss was observed, as they retained approximately 75% of the initial mass after the entire thermogravimetric analysis and presented a negligible biological compound content. To shed light on the presence of surface functional groups before and after the pyrolysis and confirming the structural changes occurred with biological structures of the plant biomass, FTIR spectra in the wavelength range of 2500 to 400 cm^−1^ are shown in Fig. 4.

FTIR results confirm similarities between pristine plant biomass and non-activated hydrochar and a strong appearance at 2300 cm^−1^ indicates the O = C = O stretching of CO_2_ caused by decarboxylation reactions observed for activated hydrochar. Around 1700–1800-cm^−1^ peaks were removed with pyrolysis treatment, so due to decarboxylation, it fits with the decrease in H/C and O/C ratios observed in the Van Krevelen diagram (Fig. 1).

Previous studies (Castro et al. 2021b; Liu et al. 2022) demonstrate that the weakening or disappearance of peaks between 1640 and 1412 cm^−1^ were produced by the cracking of protein, cellulose, and hemicellulose for hydrochars which are mostly degraded when hydrochar is pyrolyzed at 500 °C. Peaks between 1400 and 1350 cm^−1^ were caused by the C–H deformation in hemicellulose and cellulose. Lastly, peaks between 1150 and 950 cm^−1^ show the C–O bond breakdown of C–O–R from ethers and polysaccharides, catalyzed by temperature rise (Castro et al. 2021b; Tu et al. 2021; Liu et al. 2022).

To continue with structural characterization, specific surface area and microporosity of the selected hydrochar materials were evaluated using CO_2_ adsorption isotherms at 273 K.

**Fig. 5:**
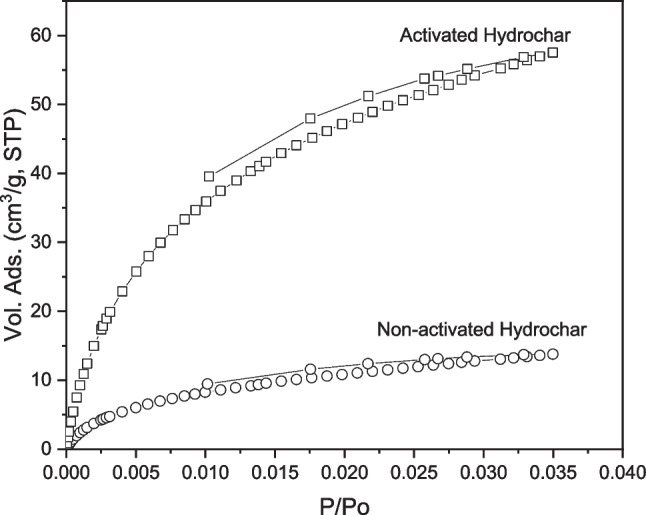
Adsorption isotherms of non-activated and activated hydrochar in CO_2_ at 273 K

The equilibrium CO_2_ adsorption/desorption isotherms at 273 K for different samples are shown in Fig. 5. As shown in this figure, the shapes of the isotherms and the amount of gas adsorbed differed significantly between the non-activated and activated hydrochars after pyrolysis. CO_2_ isotherms were used to identify narrow micropores, due to the low relative pressure that can be applied with CO_2_ at atmospheric pressure (*P*/*P*_o_ ≈ 0.035); however, this method was chosen with the aim of determining the structural differences, in terms of microporosity, of the obtained hydrochars. To calculate the main parameters describing the porosity of the obtained materials, such as the volume of micropores, micropore size, and specific surface area, the Dubinin-Radushkevich (DR) method was used according to the literature (Stoeckli et al. 2001). The parameters obtained from the DR method as well as the carbonization yield are presented in Table 1, where *E*_0_ is the characteristic energy of the adsorbate, *L* is the pore diameter, *W*_0_ is the specific volume of the micropore, *σ* is the electrical conductivity, and *S* is the specific surface area of the micropores.

**Table 1:** Main textural parameters obtained from CO_2_ adsorption isotherms for materials synthesized

	*E*_0_ (kJ/mol)	*L* (nm)	*W*_0_^*^ micropores (cm^3^ g^−1^)	*S* micropores (m^2^ g^−1^)	*σ* (S m^−1^)	Carbonization yield (%)
Non-activated hydrochar	29.00	0.61	0.04	142	0	48.25
Activated hydrochar at 500 °C	26.87	0.70	0.28	811	1.187	35.81

^*^Evaluated using DR method

Table 1 and Figures S3–S5 show an increase in the specific surface area and volume of the micropores of the non-activated and pyrolyzed hydrochar at 500 °C, which is consistent with the shape of the isotherms and thermogravimetry results. The porosity of the obtained materials is of crucial interest because the porous structure serves as a gas channel to promote the mobility and accessibility of reagents (Kitagawa 2015). Additionally, the pore size obtained by applying the DR equation was less than 2 nm (0.61 and 0.7 nm), indicating that the microporosity range does not change significantly when increasing the temperature during the activation process. Regarding electrical conductivity (*σ*), the increase experienced after pyrolysis of the hydrochar led to lower ohmic losses in the obtained material, which is expected to be relevant when it is used as an electrode material in MFCs. However, as the thermogravimetric analysis progressed the carbonization yields decreased. This can be explained by the fact that the decomposition of components was more intense and affected more structural components of the hydrochar as the thermogravimetric analysis progressed. Once the obtained hydrochar materials were characterized, they were used to dope the CF electrodes used as anodic electrodes in the MFC. The MFC performances during operation with different anodes were studied and compared.

### MFC performance

**Fig. 6:**
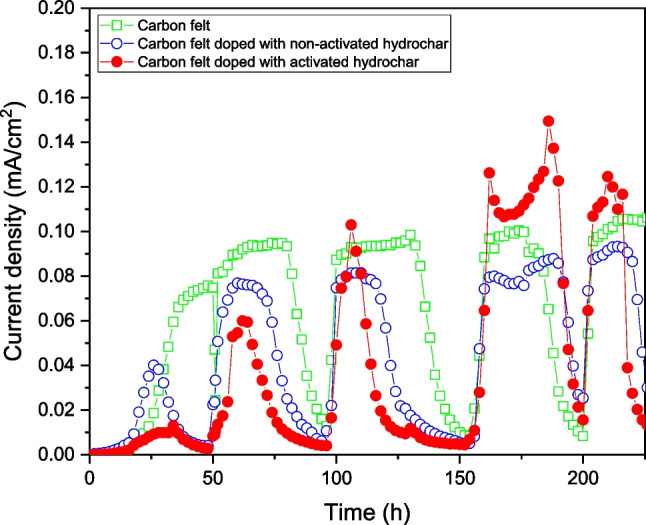
Current density generation during the start-up stage of the MFCs

The MFC performance was monitored online and recorded during the start-up stage of the MFC. Figure 6 shows the results obtained during the start-up stage.

As shown in Fig. 6, initially, the best performance was obtained using an MFC configured with a raw CF anodic electrode. During the first few hours of the start-up stage, the electrodes doped with non-activated and activated hydrochar exhibited lower electrochemical performance than that of the raw CF electrode. However, the performance of the doped electrodes drastically improved in the subsequent hours. After approximately 100 h of operation, the CF electrode doped with the activated hydrochar exhibited the highest current density, producing up to 0.11 mA/cm^2^. This value is significantly higher than that of the raw CF electrodes, which generated a current density 25% lower, 0.09 mA/cm^2^. This electrochemical performance was maintained for several hours, indicating that the performance of the raw CF anode was enhanced when it was doped with activated hydrochar. The performance of the CF electrodes doped with non-activated hydrochar was very similar to that of the raw CF electrode.

**Fig. 7:**
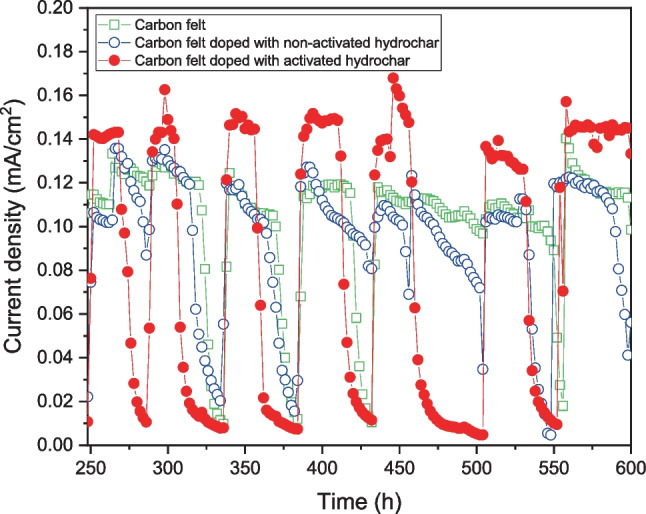
Current density generation during steady-state operation of MFC

Once the start-up stage was completed, the electrochemical performance during steady-state operation was monitored online and recorded. Figure 7 shows the steady-state current densities of the different MFCs used in this study.

As can be seen in Fig. 7, the raw CF and the CF electrode doped with non-activated hydrochar presented almost identical current density generation throughout the steady-state operation, with their values being approximately 0.12 ± 0.01 mA/cm^2^ and 0.13 ± 0.01 mA/cm^2^, respectively. It is also important to highlight that the raw CF and the CF electrodes doped with non-activated hydrochar MFCs exhibited very similar current density trends with almost identical cycles. In the case of the MFC used in this study, the anodic bioelectrochemical oxidation of the organic substrate by electrogenic microorganisms can be considered the controlling stage (Mateo et al. 2018b). This is because anodic microbial catalysis occurs at a lower rate than chemical catalysis at the cathode. Therefore, the similar cycles observed can be explained by the similar bioelectrochemical interaction between the electrogenic microbial culture and raw CF or CF doped with non-activated hydrochar anodic electrodes. In Fig. 7, it can also be observed that the highest current density produced was obtained when operating the MFC configured with the CF electrode doped with activated hydrochar, with its average current density being approximately 0.15 ± 0.004 mA/cm^2^. This current density was significantly higher (approximately 25% higher) than that obtained when operating with raw CF and CF doped with non-activated hydrochar anodes. It should also be highlighted that doping with activated hydrochar yielded a more stable current density production and reduced the cycle length. The shorter cycle length can be explained by the enhancement of the anodic bioelectrochemical oxidation rate of the organic substrates when operating the MFC configured with the CF electrode doped with activated hydrochar. Accordingly, to the results previously presented and to the literature, the increase in the anodic oxidation reaction rate can be explained by the enhanced electron transfer to the electrode due to its graphitic structure (Gonzalez del Campo et al. 2013; Mateo et al. 2016). This enhancement also allowed for more stable current density generation, avoiding fluctuations, whereas the electrodes of the raw CF and CF doped with non-activated hydrochar presented more variable current density generation. Regarding the fluctuations in the current density generation, that of the MFC operating with CF doped with activated hydrochar, measured as standard deviations, was approximately 0.005 mA/cm^2^. However, the standard deviation of the MFC operating with raw CF or CF doped with non-activated hydrochar was approximately 0.01 mA/cm^2^, a value double that obtained when operating the MFC configured with the CF electrode doped with activated hydrochar.

**Fig. 8:**
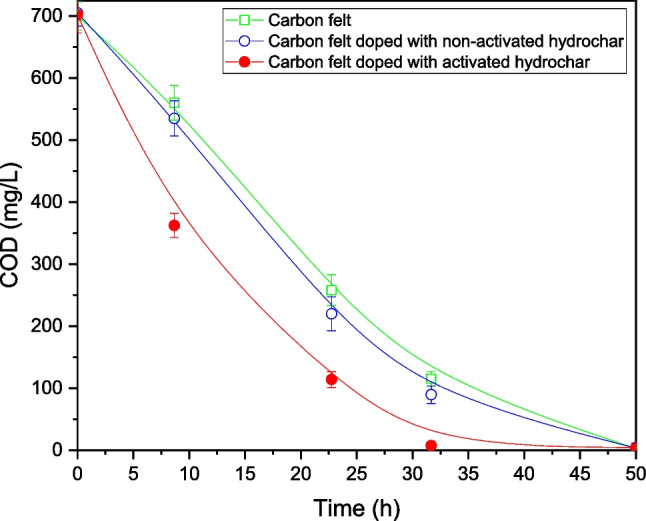
COD removal of the three MFCs during the steady-state operation

With the aim of studying the anodic fuel oxidation, the COD concentration in the anodic compartment was measured. The results obtained during a steady-state cycle are shown in Fig. 8.

As shown in Fig. 8, the highest COD removal rate was obtained when the MFC was configured with the CF anode doped with activated hydrochar. The maximum COD removal rate obtained using this device was 15 mg COD/(L·h). For the MFC configured with raw CF and CF doped with non-activated hydrochar anodes, the maximum COD removal rates were approximately 10 mg COD/(L·h). These results are in agreement with the current densities exerted by the MFCs, which correspond to the highest COD removal rates and highest current density production (Gonzalez del Campo et al. 2013; Mateo et al. 2016). Moreover, the raw CF and CF doped with non-activated hydrochar presented very similar current densities and COD removal rates. This result confirms the negligible influence of non-activated hydrochar on the performance of the raw CF electrode. From the COD removal rates and current density generation data, the steady-state CE was determined. The highest CE (35%) was obtained when the anodic electrode of the MFCs was doped with activated hydrochar. However, the CE was significantly lower (25% and 22%, respectively) than those when operating with the non-activated hydrochar and raw CF anodes, respectively. These results confirm the enhanced electrochemical performance upon doping with activated hydrochar.

**Fig. 9:**
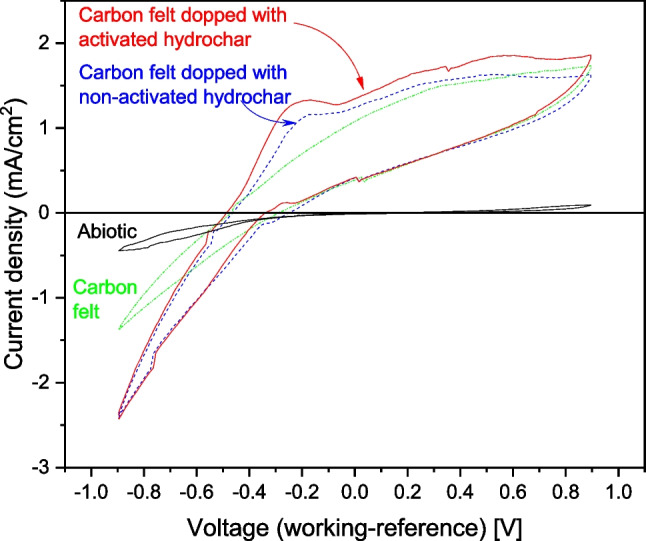
Cyclic voltammetry during steady-state operation of the MFC. Reference electrode Ag/AgCl

To characterize the electrochemical performance of the anodes used in this study, cyclic voltammetry (CV) tests were conducted. Figure 9 illustrates the results obtained during the characterization of various anodes, including the abiotic microbial fuel cell (MFC), carbon felt (CF), CF doped with non-activated hydrochar, and CF doped with activated hydrochar.

As depicted in Fig. 9, the abiotic MFC exhibited negligible electrochemical activity, as evidenced by the low measured current density. This minimal current density can be attributed to the redox reactions or redox pair in the carbon surface of the materials. The lack of substantial activity in the abiotic MFC supports the conclusion that current generation was solely driven by the microbial culture and not by any chemical reactions.

Turning to the biotic anodes, the cyclic voltammetry of the raw CF anode revealed a significant current density, noteworthy for its absence of discernible peaks in the cyclic spectrum. Conversely, when operating the MFC with the CF electrode doped with activated hydrochar, a distinct peak in the anodic potential emerged at approximately − 0.2 V vs. Ag/AgCl, accompanied by a higher current density compared to the other electrodes. According to the literature, the enhanced electrochemical performance of CF electrodes doped with activated hydrochar can be attributed to the heightened efficiency of electron transfer due to its graphitic structure (Zhang et al. 2014; Cai et al. 2020), which, in turn, improved porosity and electrical conductivity, thereby reducing mass transfer limitations and ohmic losses within these materials. Consistent with these properties, numerous energy applications of activated hydrochar as components of supercapacitors have been documented in the literature (Ding et al. 2013; Xie et al. 2023). Finally, the CF electrode doped with non-activated hydrochar exhibited intermediate behavior between those of the previous two electrodes. This intermediate behavior was similar to that of the CF electrode doped with activated biochar when operating at low voltages and similar to that of the raw CF electrode when operating at high voltages.

**Fig. 10:**
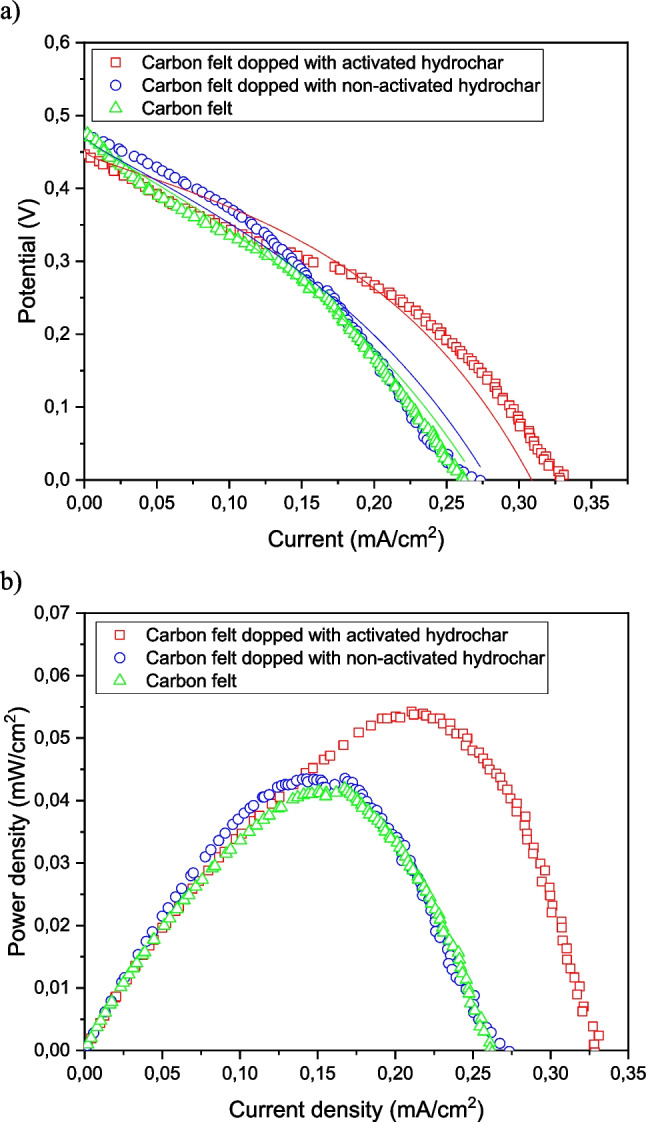
**a** Polarization lines correspond to model fitting and **b** power curves after reaching steady-state operation

Polarization and power curves were obtained to characterize the electrochemical performance of the entire MFCs. The results obtained from the polarization and power curves are shown in Fig. 10.

As shown in the polarization curves in Fig. 10(a), two different behaviors were observed. One behavior was defined by the MFC operating with the CF anode doped with activated hydrochar, and the other was defined by the MFC operating with the raw CF and CF doped with non-activated hydrochar anodes. In all cases, the ohmic loss region appeared to be the most important electrochemical loss. This can be explained by the fact that the activation and mass transport limitation losses were masked by the high resistance of the electrodes, which is more important in the cases of the CF and CF doped with non-activated hydrochar (de los Ángeles Fernandez et al. 2016). In the case of the MFC operating with the CF doped with an activated hydrochar anode, the ohmic losses seemed to have lower values, which is related to its higher porosity and electrical conductivity of the graphitic structure of the CF doped with activated hydrochar anode (see Table 1).

From the power curves presented in Fig. 10(b), it can be observed that the best electrochemical performance was obtained for the MFC configured with CF doped with an activated hydrochar anode.

Table 2 summarizes the main results obtained from the polarization curves when operating under steady-state conditions with the different substrates studied.

**Table 2:** Main electrochemical parameters at the steady state for the MFCs working with different anodes

Electrode	OCV (V)	*j*_max_ (mA cm^−2^)	*P*_max_ (mW cm^−2^)
Carbon felt	0.47	0.2624	0.041
Carbon felt + hydrochar	0.47	0.2734	0.043
Carbon felt + activated hydrochar	0.46	0.3310	0.056

From the power curves, the good performance of the MFC operated with activated hydrochar anodes can be highlighted. In all the cases the OCV was similar; however, the MFC doped with activated hydrochar reached maximum current density of approximately 0.331 mA cm^−2^. The maximum power density obtained was 0.054 mW cm^−2^, obtained at a current density of 0.22 mA/cm^2^. These values are about 25% higher than those obtained with the raw CF and the CF anodes doped with non-activated hydrochar. The lower range of intensities observed for CF and CF doped with non-activated hydrochar was due to very high ohmic losses. This can be explained by the fact that the slope of V/I was much lower in the CF doped with activated hydrochar than in the CF and CF doped with non-activated hydrochar. Finally, in the literature, enhanced electrochemical performance has been observed when operating hydrothermal carbon electrodes in sodium-ion batteries (Xie et al. 2019), which is consistent with the results obtained in this study.

With the aim to deep into the electrochemical behavior of the systems, and to quantify the relevance of the main driving forces of the electrochemical performance of the MFCs studied, the power curves were modeled by using an empirical polarization curve model described by Eqs. (1) and (2). More information about this model can be found elsewhere (Kim et al. 2007).

1 $${E}_{cell}={E}_{0}-b\cdot {\text{log}}\left(i\right)-R\cdot i-m\cdot {\text{exp}}(ni)$$

In this equation, the parameter ${E}_{0}$ can be described as where *b* log(*i*) represents the activation losses, *R*_*i*_ are the ohmic losses, and *m*·exp(*ni*) is an empirical term that approximates mass transfer loss.

2 $${E}_{0}={E}_{OCV}+b\cdot {\text{log}}({i}_{0})$$

Results from modeling the polarization curves are shown in Table 3 and lines in Fig. 10(a). As can be seen in Fig. 10(a), an accurate fitting was obtained.

**Table 3:** Fitting values of the parameters of the empirical model corresponding to the polarization curves

Parameter	Anode		
	*CF*	*CF* + *hydrochar*	*CF* + *activated hydrochar*
${E}_{0}$*(V)*	0.47	0.47	0.45
*b (V dec*^*−1*^*)*	− 0.005	− 0.005	− 0.005
${R}_{i}$*(Ω)*	− 0.92	− 0.82	− 0.39
*m (A)*	− 0.015	− 0.015	− 0.015
*n (A*^*−1*^*)*	10.0	10.0	10.0

By analyzing the results, it can be highlighted that the *E*_0_ value remained almost constant in all the cases. Regarding the *b* parameter, all the experimental results were fitted with a − 0.005 V dec^−1^ suggesting that no appreciable changes in the electrocatalytic effects can be highlighted.

The values calculated by the fitting procedure for *m* and *n* were also the same in all the cases. These results are interesting because the parameter *n* is related to the smallest current density that causes the voltage to deviate from linearity, due to mass transfer limitations. In this sense, when the current density is less than the threshold value, the term of mass transfer losses is negligible in the low and moderate current density ranges. Because of that, the constant value of *n* presented in Table 3 indicates an identical behavior in terms of mass transfer limitations in all the cases. Regarding the *m* coefficient, it is related to the relevance of the mass transfer phenomenon occurring over the entire range of current densities. As can be seen in Table 3, the *m* values were also the same in all the cases. Because of that, similar performance effects were expected when the threshold current density is overcome.

The main difference was observed in the *R*_*i*_ value, presenting the lowest value of *R*_*i*_ of the anode based on CF doped with activated hydrochar. The low value of this anode can be explained by a more conductive electrodic surface due to the contribution of the activated hydrochar. It is important to remark that depending on the nature of the electrodic material, the different electron transfer mechanisms (direct by means of cytochrome proteins or conductive pili as well as by indirect mechanisms by means of chemical mediators) are facilitated as well as the subsequent electron conduction through the electrode. Because of that, the better performance when operating with CF doped with activated hydrochar can be explained in terms of a lower *R*_*i*_ caused by the enhancement of the electron transference and transport processes taking place in the anode.

## Conclusions

Based on the results obtained in this study, several important conclusions can be drawn. Initially, it was observed that the start-up phase of the microbial fuel cell (MFC) utilizing a raw CF anode was faster in comparison to MFCs employing CF anodes doped with non-activated or activated hydrochar. However, as the MFCs reached a steady-state operation, the MFC configured with CF anodes doped with activated hydrochar consistently demonstrated higher electrochemical performance compared to those configured with raw CF and CF doped with non-activated hydrochar anodes. The better performance of the activated hydrochar was explained by the enhanced electron transference of the highly graphitic carbonaceous structures obtained during the activation procedure at 500 °C as obtained in the XRD analysis.

The maximum power output achieved by the MFC equipped with CF doped with activated hydrochar anodes was approximately 60 µW/cm^2^, while the power generated by MFCs featuring raw CF and CF doped with non-activated hydrochar anodes was approximately 40 µW/cm^2^. This represents a notable 30% increase in power output. In general, the results indicate that the activation losses, kinetics limitation, and mass transfer seemed to be very similar in all the anodes, being the main difference the ohmic losses which were reduced in the Cf doped with activated hydrochar due to its graphitic structure.

The enhanced electrochemical performance of the CF anode doped with activated hydrochar holds significant importance for two main reasons. Firstly, it enables the potential for higher energy generation within MFCs, promising more efficient and sustainable energy production. Secondly, harnessing electrical energy from anodes created through the valorization of plant biomass waste materials aligns with the principles of the circular economy, where resources are conserved, and wastes are transformed into valuable products, contributing to a more sustainable and eco-friendly approach.

## Supplementary Information

Below is the link to the electronic supplementary material.

Supplementary file1 (DOCX 3019 KB)

## Data Availability

Data available on request from the authors.

## Abbreviations

COD: Chemical oxygen demand
CF: Carbon felt
CFnaH: CF electrode doped with non-activated hydrochar
CFaH: CF electrode doped with activated hydrochar
rCOD: COD removal rate
CE: Coulombic efficiency
DR: Dubinin-Radushkevich
HTC: Hydrothermal carbonization
MFC: Microbial fuel cell
OCV: Open-circuit voltage
