# Supplementary material for: Effect of hydrochar-doping on the performance of carbon felt as anodic electrode in microbial fuel cells
Source: Environ Sci Pollut Res Int. 2024 Apr 23;32(49):28253–65. doi: 10.1007/s11356-024-33338-2 (PMC12712122; doi:10.1007/s11356-024-33338-2)
Supplement: Supplementary file 1 — Supplementary file1 (DOCX 3019 KB) [file 11356_2024_33338_MOESM1_ESM.docx]

Effect of hydrochar-doping on the performance of carbon felt as anodic electrode in microbial fuel cells

*Yelitza Delgado^1^, Natalia Tapia^1,2^, Martín Muñoz-Morales^1^, Álvaro Ramirez^1^, Javier Llanos^1^, Ignacio Vargas^2^, Francisco Jesús Fernández-Morales^1*^*

^1^ Department of Chemical Engineering. ITQUIMA. University of Castilla La Mancha. Campus Universitario s/n. 13071 Ciudad Real. Spain.

^2^ Department of Hydraulic and Environmental Engineering, Pontificia Universidad Católica de Chile, 7820436 Santiago, Chile.


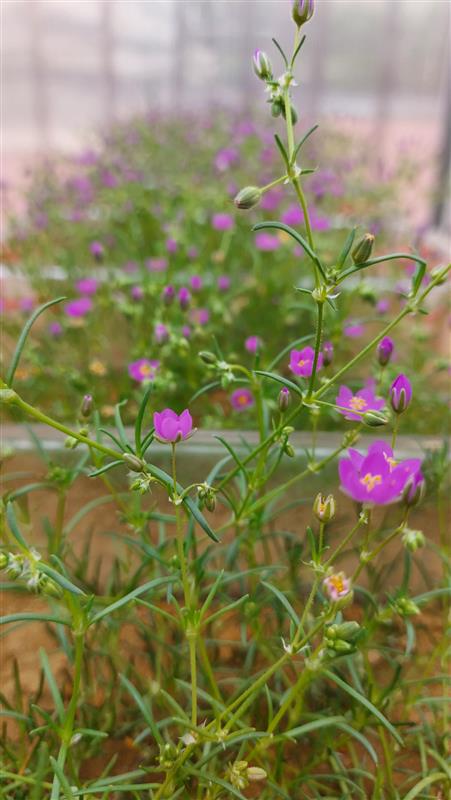


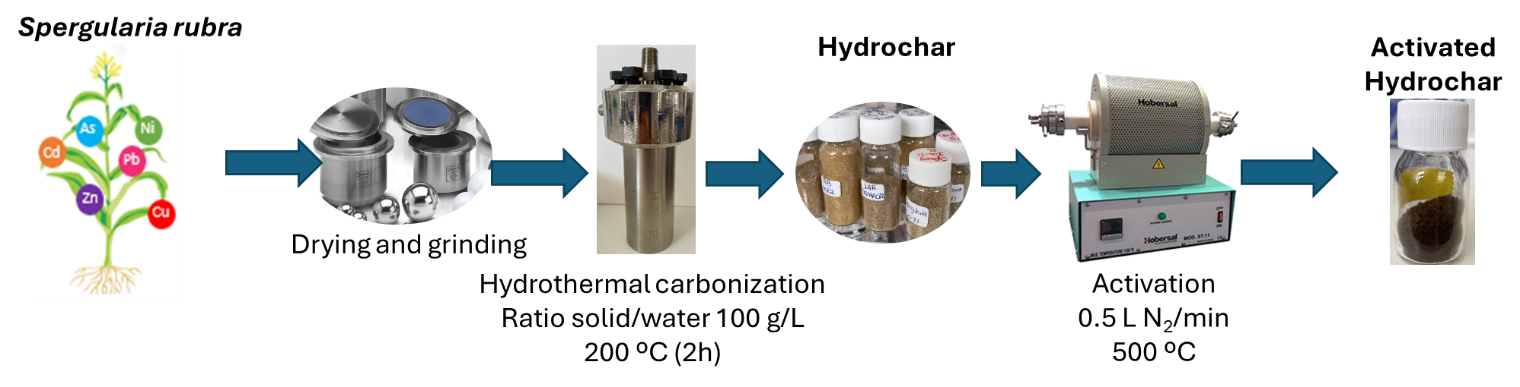


**Figure S1.** a) *Spergularia rubra. b) Transformation into non-activated hydrochar and activated hydrochar.*


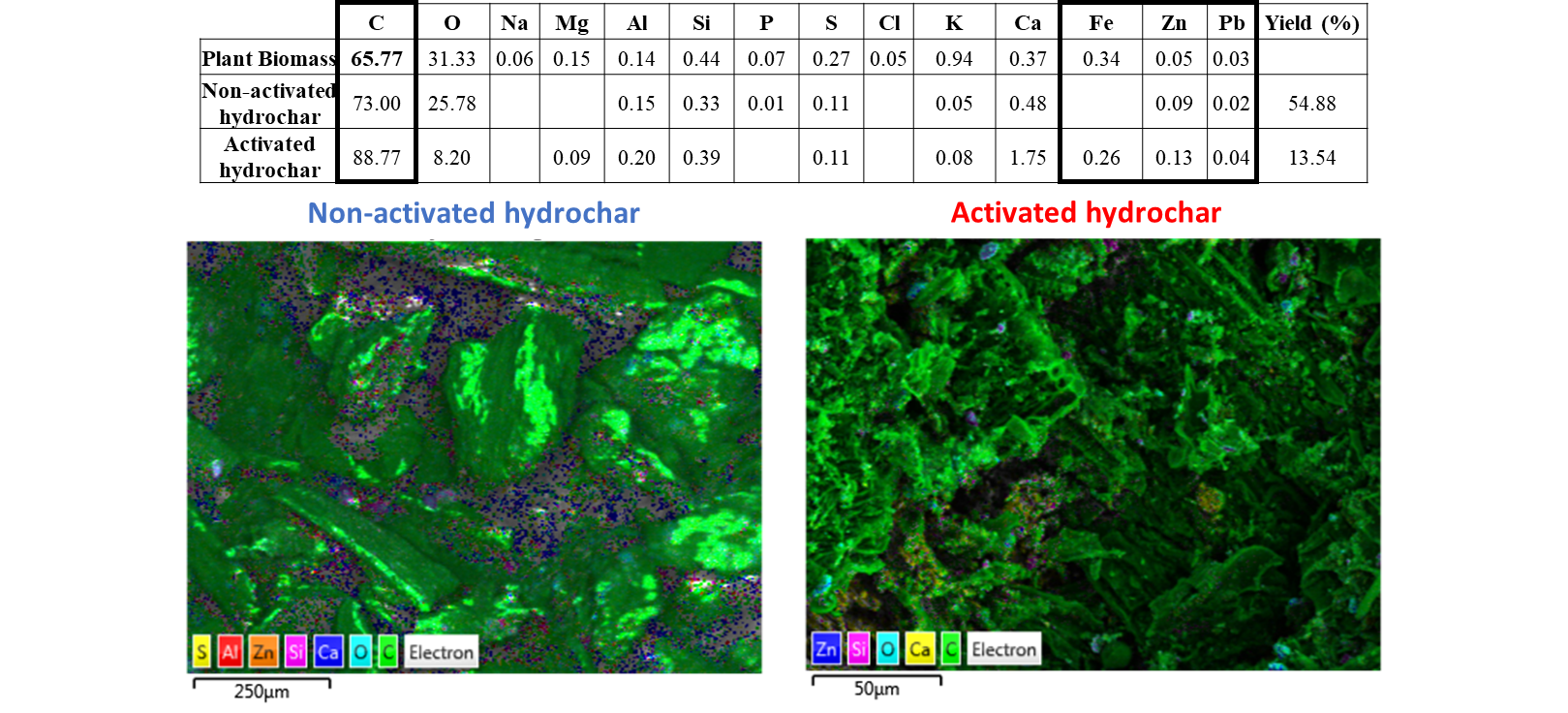


**Figure S2.** EDX mapping of main atomic composition of non-activated and activated hydrochars used with dopped carbon felt electrodes.





**Figure S3.** SEM image of the raw plant biomass.





**Figure S4.** SEM image of the non-activated hydrochar.





**Figure S5.** SEM image of the activated hydrochar.
